# PhyloBot: A Web Portal for Automated Phylogenetics, Ancestral Sequence Reconstruction, and Exploration of Mutational Trajectories

**DOI:** 10.1371/journal.pcbi.1004976

**Published:** 2016-07-29

**Authors:** Victor Hanson-Smith, Alexander Johnson

**Affiliations:** Department of Microbiology and Immunology, University of California, San Francisco, San Francisco, California, United States of America; University of Canterbury, NEW ZEALAND

## Abstract

The method of phylogenetic ancestral sequence reconstruction is a powerful approach for studying evolutionary relationships among protein sequence, structure, and function. In particular, this approach allows investigators to (1) reconstruct and “resurrect” (that is, synthesize *in vivo* or *in vitro*) extinct proteins to study how they differ from modern proteins, (2) identify key amino acid changes that, over evolutionary timescales, have altered the function of the protein, and (3) order historical events in the evolution of protein function. Widespread use of this approach has been slow among molecular biologists, in part because the methods require significant computational expertise. Here we present PhyloBot, a web-based software tool that makes ancestral sequence reconstruction easy. Designed for non-experts, it integrates all the necessary software into a single user interface. Additionally, PhyloBot provides interactive tools to explore evolutionary trajectories between ancestors, enabling the rapid generation of hypotheses that can be tested using genetic or biochemical approaches. Early versions of this software were used in previous studies to discover genetic mechanisms underlying the functions of diverse protein families, including V-ATPase ion pumps, DNA-binding transcription regulators, and serine/threonine protein kinases. PhyloBot runs in a web browser, and is available at the following URL: http://www.phylobot.com. The software is implemented in Python using the Django web framework, and runs on elastic cloud computing resources from Amazon Web Services. Users can create and submit jobs on our free server (at the URL listed above), or use our open-source code to launch their own PhyloBot server.

“This is a *PLOS Computational Biology* Software paper.”

## Introduction

Over the last decade, several innovative studies analyzed evolutionary trajectories of ancient genes in order to discover important relationships between present-day gene sequence, structure, and function [1–6]. These discoveries relied on the methods of ancestral sequence reconstruction, in which models of amino acid evolution are used to infer ancient protein sequences at multiple points in a gene family history [7]. Ancestral proteins have been “resurrected” in several cases [8]; that is, they have been expressed in living cells deleted for the modern descendant and purified and studied *in vitro*. Comparisons with the modern counterparts led to the discovery of key amino acid residues responsible for the biochemical diversity among related members of a gene family (for a review see [9]). The method also allows the evolutionary path to a modern protein to be accurately reconstructed, illustrating how “permissible” trajectories circumvent fitness barriers and produce novelty. This analysis is not possible without ancestral reconstruction.

Many questions in molecular and cell biology could be addressed using ancestral protein analysis. One obstacle is that the typical protocol for ancestral reconstruction involves multiple steps that require significant expertise with computational phylogenetics. In brief, the protocol begins with a collection of orthologous protein sequences sampled from diverse species. Next, the sequences are aligned to each other, their phylogenetic relationships are inferred, probabilities of ancestral sequences are computed at internal phylogenetic nodes, and then mutations (which covert ancestral to modern proteins, or vice versa) are identified on every phylogenetic branch. The rigorous application of this protocol can be challenging because it is not implemented as a single software package. Rather, ancestral reconstruction currently requires dozens of software tools, the computational skills to combine them, knowledge about phylogenetic models, and the programming abilities to deal with multiple file formats (many of them esoteric).

PhyloBot, described here, is new software that automates ancestral sequence reconstruction. It provides a user interface that greatly simplifies the reconstruction process, and also includes visual tools to analyze ancestors. Specifically designed for bench scientists unfamiliar with bioinformatics, the software runs in web browsers and it requires no installation on users’ computers. Rather, PhyloBot uses elastic computing resources in the Amazon cloud. Moreover, results from PhyloBot analyses are portable: every ancestral reconstruction receives a permanent URL that can be shared with colleagues and used in publications. We believe PhyloBot is a significant methodological advance for computational molecular biology, one that will hopefully inspire widespread use of ancestral protein analysis.

## Design and Implementation

**Fig 1:**
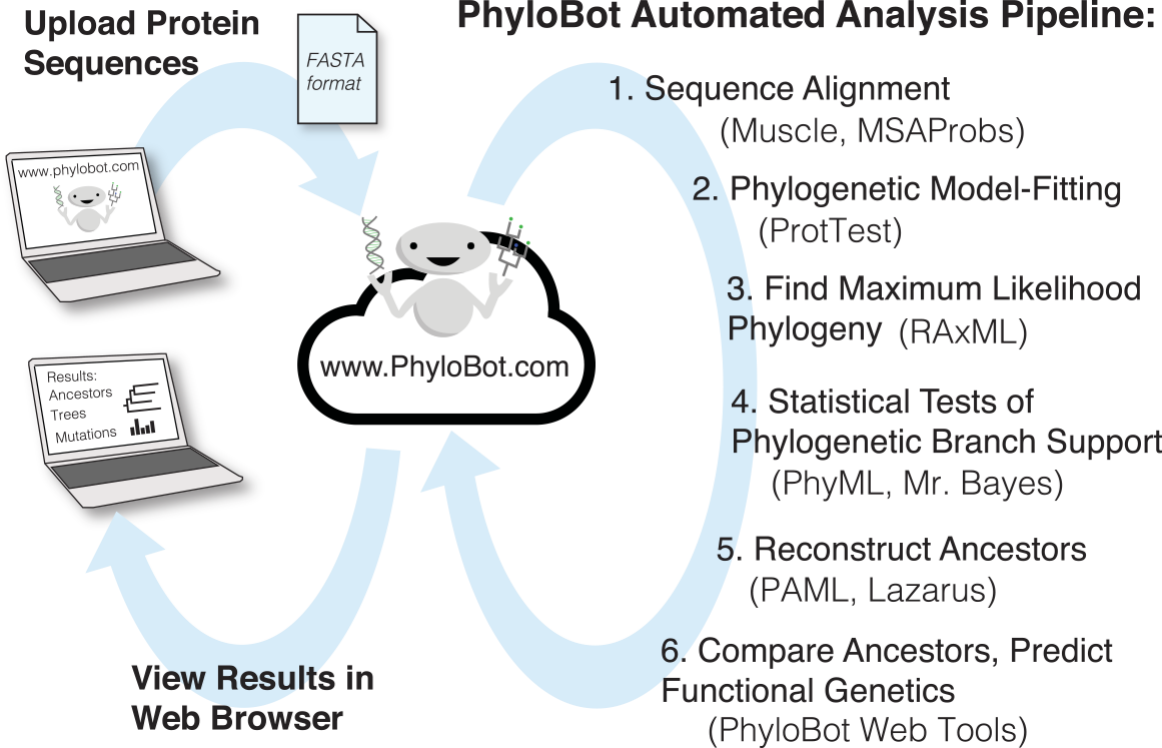
Summary of PhyloBot automated pipeline. A user begins by uploading a collection of orthologous protein sequences in a FASTA-formatted text file. PhyloBot reads the sequence collection and launches its automated analysis pipeline, which includes sequence alignment, phylogenetic model-fitting, tests of branch support, ancestral sequence reconstruction, and prediction of functional genetics. Upon completion, the results can be viewed in a web browser.

PhyloBot is a web portal that automates the reconstruction of ancestral amino acid sequences. The portal provides interactive web tools to compose and launch analysis jobs on remote supercomputers. The tools are easy-to-use and conceal a great deal of underlying automation. To start, users upload a FASTA-formatted text file containing a collection of related protein sequences (Fig 1). There is no minimum requirement for the degree of relatedness between the sequences, but in general, only conserved portions of a protein can be reconstructed accurately. For most investigations, the evolutionary trajectory of conserved regions of proteins are the principle interest. PhyloBot flows the sequences automatically through six major stages of analysis, using a dozen different software packages (Table 1). Upon completion, the results from all stages can viewed in a web browser.

**Table 1:** Software incorporated in the PhyloBot analysis pipeline. PhyloBot uses several existing software tools at various stages in its automated analysis pipeline.

Software	Purpose	Reference
MUSCLE v3.8.31	Multiple Sequence Alignment	[10]
MSAProbs 0.9 5r1	Multiple Sequence Alignment	[11]
FastTree v2.1.7	Rapid ML Tree Estimation (for ZORRO)	[12]
ZORRO	Alignment Quality Estimation	[13]
RAxML v8.1.15	ML Phylogenetic Estimation	[14]
PhyML v20130708	Phylogenetic Branch Support Estimation	[15,16]
Lazarus v2.7.6	Controlling CODEML	[17]
CODEML/PAML v4.2	Empirical Bayesian Ancestral Sequence Reconstruction	[18]
DendroPy	Manipulating Phylogenies in Software	[19]
Python Django v7	Interactive Web Tools, Server Logic	http://www.djangoproject.com
Amazon Web Services	Web Hosting	http://aws.amazon.com

**Fig 2:**
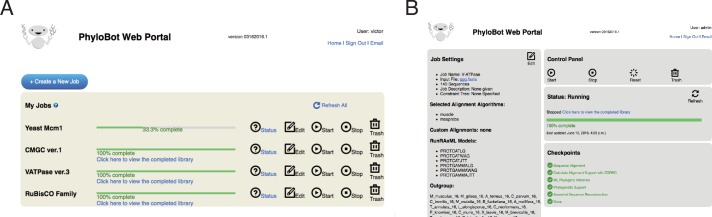
Screenshots from the PhyloBot web portal. (A) The front page of the portal provides a control panel to create new jobs and to check the status of existing jobs. In this image, a user has five jobs; three of them are 100% complete and the other two are in progress. (B) A user can view detailed status for every job they create. The status page provides controls to start, stop, reset, and delete the job, in addition to displaying the job’s settings and the job’s current status.

The front page of the PhyloBot portal provides a control panel to compose new analysis jobs (Fig 2A), and to check the status of existing jobs (Fig 2B). Composing a new job is relatively simple: a user uploads a collection of protein sequences in FASTA format, creates a unique name for the job, and specifies the “outgroup”–i.e., a group of the sequences that can be used to root the phylogenetic tree. A user can immediately launch the job using the default settings (which are appropriate for most analyses), or customize the job. The default settings will reconstruct ancestors using a collection of different sequence alignment methods and phylogenetic models. A user can optionally provide a so-called “constraint tree” that specifies evolutionary relationships among protein sequences that are assumed *a priori* to be true. If this tree is provided, PhyloBot will use it to restrict the phylogenetic analysis to evolutionary hypotheses that match the constraints.

PhyloBot is engineered using Python Django, and it currently runs on cloud computing resources from Amazon Web Services. When a job is launched, PhyloBot acquires elastic compute nodes from Amazon. This means that all jobs are launched instantly, and there is no queue to wait. Users are welcome to use an instance of PhyloBot available at http://www.phylobot.com, or launch their own instance of PhyloBot using its open-source code.

### Multiple sequence alignment

The inference of homology between sites in related protein sequences (i.e., multiple sequence alignment) is a necessary first step for phylogenetic analysis. Many alignment methods have been proposed [20, 21], and different methods can result in conflicting phylogenetic conclusions [22]. Open reading frames are inherently difficult to align, and no single alignment method has been found to be accurate in all conditions. PhyloBot uses two different methods and compares their results: Muscle [10], and MSAProbs [11]. Both methods progressively align sequences according to a guide tree. The methods differ in their approaches to estimating the guide tree, and in their approaches to estimating the costs of sequence insertions and deletions events. PhyloBot also provides a feature for users to upload their own pre-computed sequence alignments. The uploaded alignments are then used alongside the alignments computed by Muscle and MSAProbs. After sequence alignment is complete, alignment quality is estimated using a probabilistic masking method [13].

**Fig 3:**
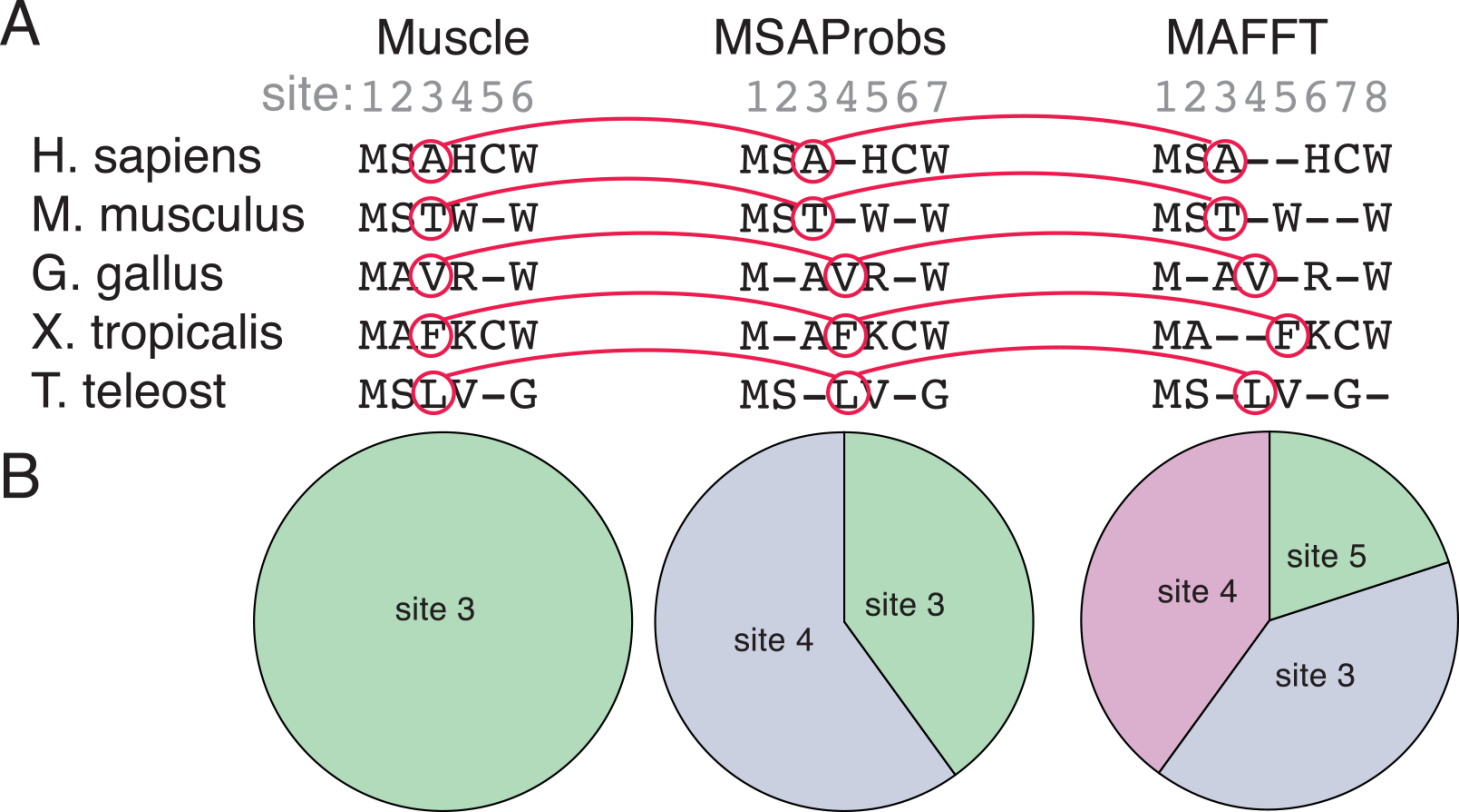
Example of alignment robustness analysis. In this simple example, orthologous amino acid sequences from five species were aligned using three different methods for multiple sequence alignment: Muscle, MSAProbs, and MAFFT. (A) PhyloBot maps the aligned position of every character across all alignments. Shown in red is the character map for the amino acids aligned into site 3 of the Muscle alignment. In the MSAProbs sequence alignment, these same residues are split across sites 3 and 4. In the MAFFT alignment, these residues are split across sites 3, 4 and 5. (B) PhyloBot displays the character map as pie charts expressing site identity relative to the Muscle alignment. PhyloBot will also show these maps relative to MSAProbs and MAFFT alignments.

PhyloBot evaluates the consistency of sequence alignments by mapping the aligned position of every residue to its corresponding position in other alignments (Fig 3A). This comparison reveals the extent to which an inferred “site” in one alignment may be one, two, or multiple sites in another alignment (Fig 3B). These differences can have significant consequences for later stages in ancestral reconstruction analysis. Specifically, the lengths of reconstructed ancestral protein sequences are determined by the number of sites in the underlying alignment. Disagreements between alignment methods, therefore, can produce ancestral sequences of different lengths. PhyloBot provides visual tools to evaluate the consistency and robustness of sequence alignments, and to rapidly examine their differences.

### Phylogenetic inference

PhyloBot infers phylogenies using a maximum likelihood (ML) method implemented in RAxML [14]. Briefly, the ML method searches for the tree and branch lengths with the highest probability of producing the sequence alignment, based on a model of amino acid substitution [22]. Many models have been proposed to account for different evolutionary patterns. For example, some models allow for heterogeneity in the evolutionary rates at different sites [23], while other models allow for heterogeneity in the amino acid substitution process at different sites [24]. PhyloBot finds the best-fitting model from a collection of options, using the Akaike Information Criterion (AIC) to measure model fit [25]. This approach, specifically the use of the AIC, is similar to the method implemented in the popular software ProtTest [26].

**Fig 4:**
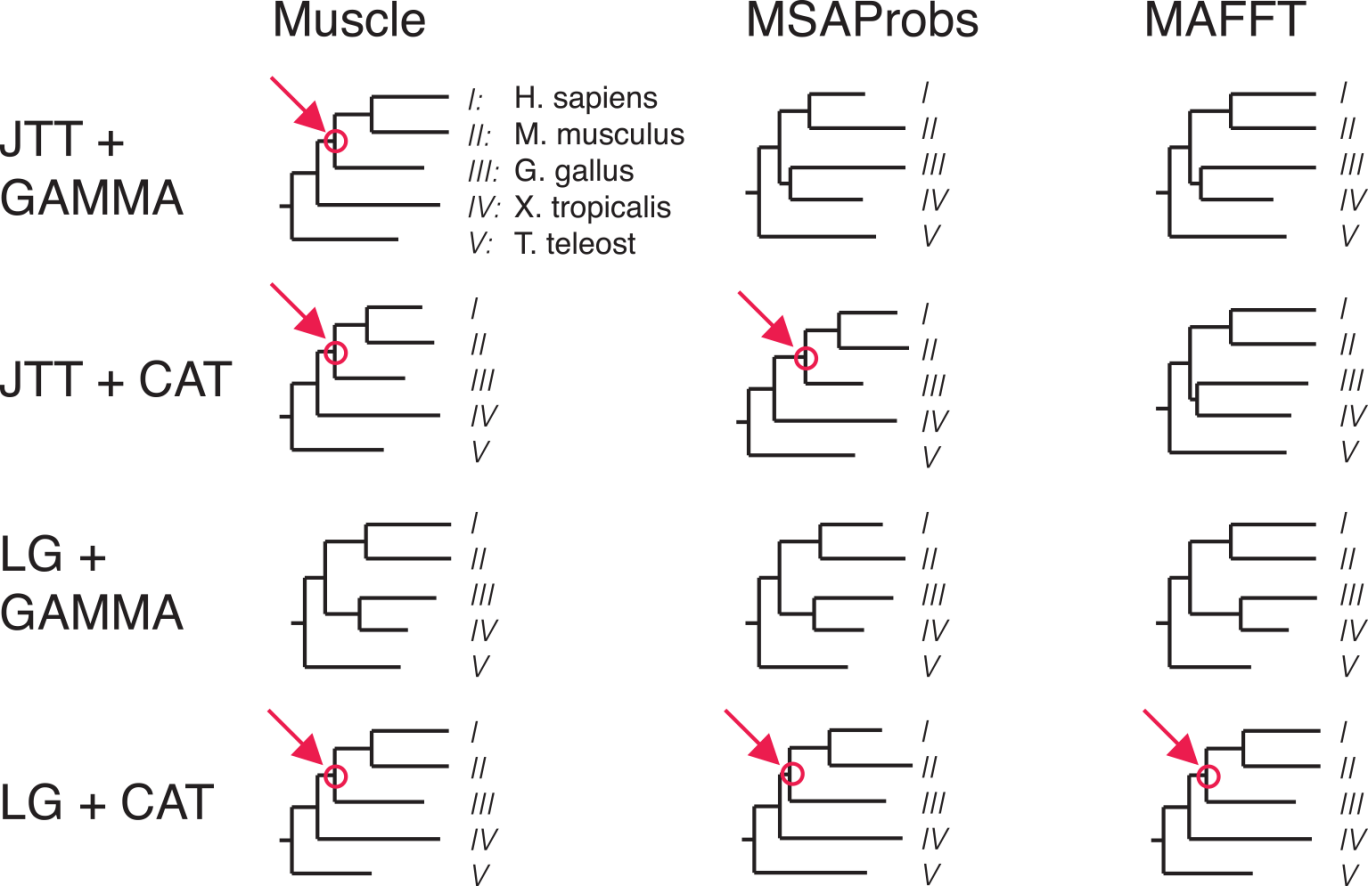
Example of ancestral node robustness analysis. In this small example with protein sequences from five species, maximum likelihood phylogenies were inferred using four different evolutionary models (JTT+GAMMA, JTT+CAT, LG+GAMMA, and LG+CAT) based on three different sequence alignment methods (Muscle, MSAProbs, and MAFFT). The resulting ML phylogenies disagree in their topologies, and an ancestral node in one tree may not exist in other trees. For example, shown in red is the phylogenetic node corresponding to the most-recent ancestor of *H*. *sapiens*, *M*. *musculus*, and *G*. *gallus*, with *X*. *tropicalis* and *T*. *teleost* as the outgroup. This ancestral node is not inferred to exist when using some combinations of models and methods. Specifically, the alternate phylogenies support an evolutionary hypothesis in which the sequences from *G*. *gallus* and *X*. *tropicalis* are sister to each other. PhyloBot gathers this information about all reconstructed ancestral nodes, in order to assess the extent to which an ancestor’s existence is robust across different models and methods.

As a consequence of the model-fitting step, PhyloBot finds ML trees for all combinations of sequence alignments and evolutionary models in its collection. This means that phylogenetic conclusion drawn from one method-model pair can be assessed for robustness across alternate methods and models (Fig 4). Different method-model combination can reveal discrepant phylogenies that affect interpretations of protein evolution. PhyloBot screens for these discrepancies by mapping every ancestral node to its corresponding node(s) on the trees found using different approaches. This type of ancestral node robustness analysis reveals those ancestors that are contingent on method and model choice; due to incompatible branching topologies, an ancestor may not exist on all trees.

The accuracy of every tree branch is estimated using approximate likelihood ratio tests (aLRT), implemented in PhyML v3.0 [16,17]. The aLRT is quick and relatively accurate compared to other confidence methods [27, 28]. For ease of interpretation, PhyloBot transforms aLRT test statistics into a simple approximate likelihood ratio (aLR) as follows: $aLR=e^{\left(\frac{aLRT}{2}\right)}$

The aLR for a particular branch can be interpreted as an estimated likelihood ratio between two different evolutionary hypotheses. In the first hypothesis, the true tree is the ML tree containing the branch in question. In the second hypothesis, the true tree is an alternate tree in which the branch does not exist. Using this framework, it can be said that the existence of specific phylogenetic split is estimated to be “X times more likely” than the next-best hypothesis in which that branch doesn’t exist.

### Ancestral sequence reconstruction

PhyloBot reconstructs ancestral protein sequences at every internal node of every ML tree, for all combinations of sequence alignment method and evolutionary model. Ancestors are reconstructed using the empirical Bayes approach [7], as implemented in the software CODEML [18]. This approach calculates a probability distribution of ancestral sequences for every ancestral node. The ML sequence for a single node can be found by concatenating the highest probability residue at each site into a string of amino acids. PhyloBot uses *Lazarus* [17] to control CODEML, and places ancestral insertion/deletion characters by parsimony [28]. Previous work suggests that ML ancestral sequences encode proteins that tend to overestimate thermostability [29]. Following from this work, PhyloBot computes a collection of Bayesian-sampled sequences that sometimes choose less-probable amino acids from the probability distribution.

### Exploration of mutational trajectories

PhyloBot provides web tools to compare ancestral protein sequences at different points in evolutionary history. Ancestral sequence comparison is a direct means to generate testable hypotheses about which residues in a protein determine its unique biochemistry. In many protein families, all members perform an analogous function, such as binding a class of substrates, but individual members exhibit biochemical variation in this function. Sequence comparisons between present-day proteins often suggest a large number of possible amino acid changes to explain observed biochemical differences. In contrast, comparisons between ancestral sequences on relevant phylogenetic branches may reveal a smaller set of candidate residues with fewer false-positives [30].

## Results

PhyloBot has been used to discover genetic mechanisms underlying biochemical diversity in several protein families, including protein kinases [4], DNA-binding transcription regulators [3], and transmembrane ion pumps [31]. In these studies, ancestral reconstructions from PhyloBot were also used to order key evolutionary steps. Interactive results from these projects can be viewed in a web browser at the following URLS: http://www.phylobot.com/cmgc, http://www.phylobot.com/mcm1, and http://www.phylobot.com/VATPase. The methods of ancestral reconstruction can be applied to nearly any protein family, regardless of its age or diversity. The accuracy of a reconstruction is correlated with conservation; this means that functionally important interaction domains are generally reconstructed with higher accuracy than poorly conserved regions, such as polypeptide linkers.

**Fig 5:**
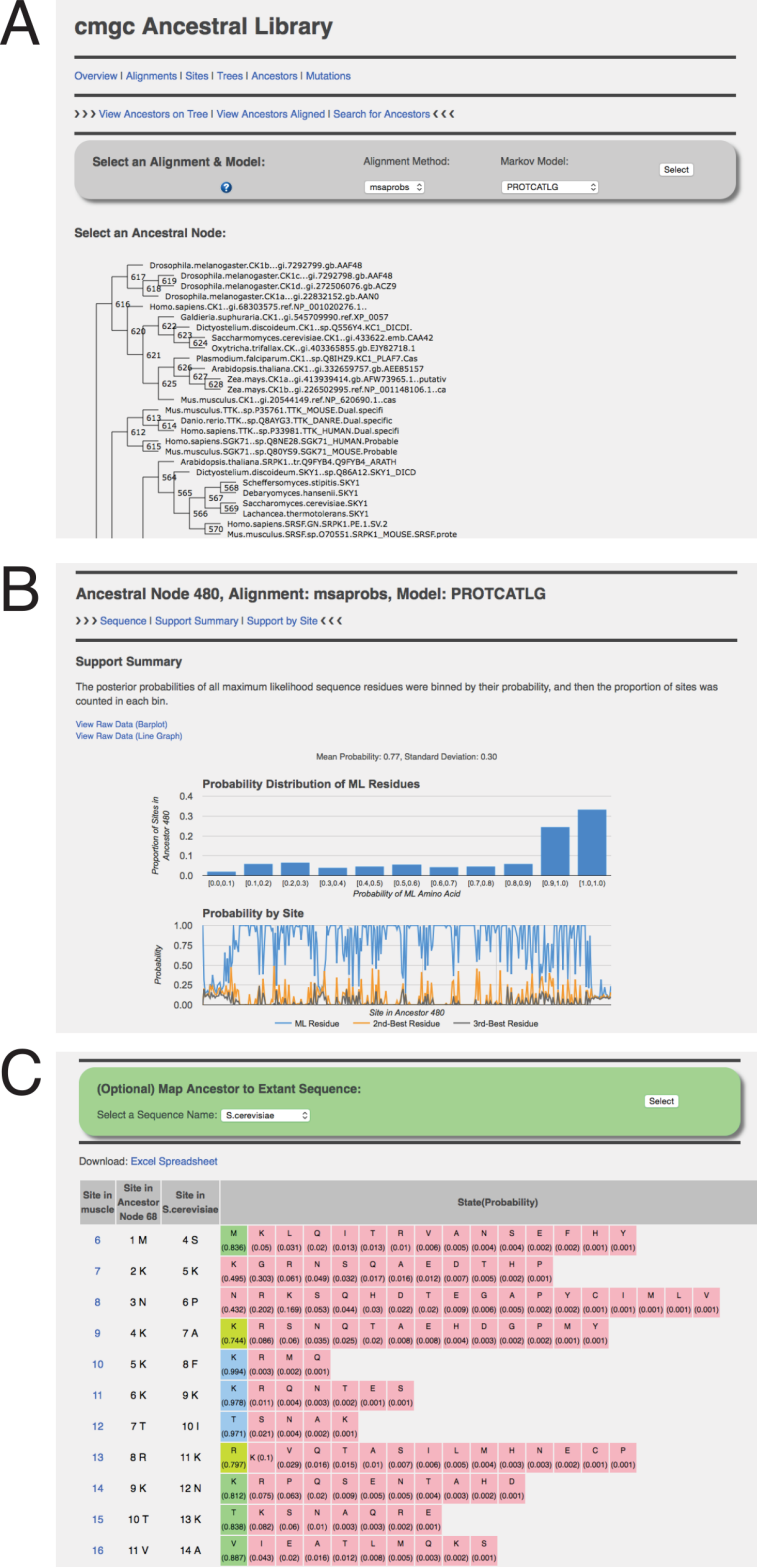
Screenshots from the PhyloBot ancestral library viewer. The images shown come from the Ancestral Library computed for the CMGC protein family [31]. (A) The library viewer displays an interactive tree for exploring reconstructed protein ancestors. Users select the maximum likelihood tree based on the alignment method and evolutionary model, and then click on ancestral nodes within that tree. (B) PhyloBot gathers summary statistics about every ancestral node. Shown here is the support summary for ancestral Node 401 in the CMGC family, reconstructed using msaprobs and PROTCATLG. The histogram bins the sequence sites of Node 401 according to their amino acid probability support. In this case, a majority of sites have support of 0.9 or greater. The line graph expresses the probability of the maximum likelihood amino acid residue, along with the second-best and third-best reconstructed residues; the line graph is a quick way to visually determine which protein domains were reconstructed with strong support. In this example, there is an unstructured region in the C-terminus that was reconstructed with low support. (C) PhyloBot shows details about every site in every reconstructed ancestor. Shown here is the probability support by site for Node 401 in CMGC. Users can optionally map this data to extant sequences. For example, here a user selected Homo sapiens CDK6. In the table the first column displays the sequence site in the MSAProbs alignment, the second column expresses the site number and best amino acid state in the reconstructed ancestor Node 401, the third column expresses the site number and amino acid state in Homo sapiens CDK6, the fourth column expresses the full probability distribution of all amino acid states reconstructed at that site in Node 401.

PhyloBot provides an ancestral library viewer to interact with results from completed analyses (Fig 5). In practice, PhyloBot deduces from modern protein sequences the ancestral sequences, expressed as probabilities of a given amino acid at any branching point in the phylogenetic tree. In many cases, the probability is sufficiently high that the ancestral protein can be “resurrected” with high accuracy. Every ancestral library gets a unique URL, which is permanent and can be shared with collaborators, or anyone else interested in viewing the ancestors. Users register for an account with PhyloBot, and analyses submitted by a particular user are visible only by him/her unless the analysis URL is shared. The ancestral viewer displays results from all stages of the PhyloBot analysis: sequence alignments, trees, ancestors, statistical support, and mutations on branches.

The methods of ancestral reconstruction are ideal for examination of protein families with one or more diverse biochemical functions that can be assayed in molecular experiments. In these cases, PhyloBot is well-suited to guide experimentalists toward identification of the residues that determine functional variation across a protein family.

## Availability and Future Directions

PhyloBot is available to use at http://www.phylobot.com, and its source code is available at https://github.com/vhsvhs/phylobot-django. Future versions of PhyloBot will include an expanded suite of alignment methods and phylogenetic models.
